# Mechanisms through which exercise reduces symptom severity and/or functional impairment in posttraumatic stress disorder (PTSD): Protocol for a living systematic review of human and non-human studies

**DOI:** 10.12688/wellcomeopenres.19903.3

**Published:** 2025-04-14

**Authors:** Simonne Wright, Toshi A. Furukawa, Malcolm Macleod, Ouma Simple, Olufisayo Elugbadebo, Virginia Chiocchia, Claire Friedrich, Edoardo G. Ostinelli, Jennifer Potts, Fiona J. Ramage, Spyridon Siafis, Claire Stainsfield, Francesca Tinsdeall, James Thomas, Andrea Cipriani, Georgia Salanti, Soraya Seedat, the GALENOS team

**Affiliations:** 1 South African Medical Council Unit on the Genomics of Brain Disorders, Department of Psychiatry, Stellenbosch University, Stellenbosch, Western Cape, 7500, South Africa; 2 Department of Health Promotion and Human Behavior, Graduate School of Medicine / School of Public Health, Kyoto University, Kyoto, Kyoto Prefecture, 606-8303, Japan; 3 Centre for Clinical Brain Sciences, The University of Edinburgh, Edinburgh, Scotland, EH8 9YL, UK; 4 School of Medicine, College of Health Sciences, Makerere University, Kampala, Central Region, 8HQG+FPF, Uganda; 5 College of Medicine, University of Ibadan, Ibadan, Oyo, 200132, Nigeria; 6 Institute of Social and Preventive Medicine, University of Bern, Bern, Canton of Bern, 3012, Switzerland; 7 Oxford Precision Psychiatry Lab, NIHR Oxford Health Biomedical Research Centre, University of Oxford, Oxford, England, OX3 7JX, UK; 8 Department of Psychiatry, University of Oxford, Oxford, England, OX3 7JX, UK; 9 Department of Psychiatry and Psychotherapy, School of Medicine, Technical University of Munich, Munich, Bavaria, 81675, Germany; 10 EPPI Centre, Social Research Institute, University College London, London, England, WC1H 0NS, UK; 11 Oxford Health NHS Foundation Trust, Warneford Hospital, Oxford, England, OX3 7JX, UK

**Keywords:** GALENOS, PTSD, exercise, extinction learning, memory regulation, emotion regulation

## Abstract

**Background:**

Exercise can play an important role in reducing symptom severity and improving functional impairment in patients with posttraumatic stress disorder (PTSD). However, the precise mechanisms underpinning the effect of exercise in PTSD management are not fully understood. This living systematic review aims to synthesize and triangulate the evidence from non-human and human studies to gain insight into the biopsychosocial mechanisms through which exercise reduces symptom severity and functional impairment.

**Methods:**

Independent searches will be conducted in electronic databases to identify eligible studies. Two reviewers will independently conduct the study selection, data extraction, and risk of bias assessment. We will extract outcome data and variables that can act as effect modifiers or as mediators of the effect of exercise. For the non-human studies, outcome data will include the non-human equivalents of PTSD symptom clusters. For human studies, the primary outcome will be PTSD symptom severity. The secondary outcomes will be avoidance symptom severity, reexperiencing symptom severity, hyperarousal symptom severity, negative cognitions and mood severity, functional impairment, loss of PTSD diagnosis, and dropout rates.

To explain the biopsychosocial mechanisms through which exercise affects the outcome of interest, we will extract effects that relate to the impact of exercise on potential mediating variables and the effect of the later outcomes. Comparison of within-study direct and indirect effects obtained from mediation analysis, when reported, will provide insight into the importance of the examined mediator.

If appropriate, we will synthesize study results using meta-analyses. We will examine potential effect modifiers of the total exercise effect to understand better the impact of exercise on PTSD symptoms and function impairment (when possible). The evidence about the potential mediators of the association between exercise and PTSD-related outcomes will be considered in a consensus meeting when sufficient evidence is available.

**Protocol registration:**

PROSPERO-ID: 453615

## Introduction

### Background

Posttraumatic stress disorder (PTSD) is a trauma-related disorder that develops in response to a traumatic event ( American Psychiatric Association, 2013). It is primarily characterized by (i) reexperiencing symptoms, reliving the traumatic event in the form of flashbacks and nightmares, (ii) avoidance symptoms, a deliberate effort to avoid reminders of the traumatic event/s, (ii) negative cognitions and mood, and (iii) hyperarousal symptoms, such as irritability, aggression, risky or destructive behaviour, hypervigilance, heightened startle reaction, and difficulty concentrating and sleeping ( American Psychiatric Association, 2013). Furthermore, it is common for individuals with PTSD to suffer from psychiatric comorbidities including anxiety, depression, substance use disorder, and insomnia ( Goldstein *et al*., 2016; Whitworth *et al*., 2022). While 25–40% of PTSD cases can be expected to remit within one year, for many these symptoms can endure indefinitely ( Bromet *et al*., 2017). Many psychological treatments have demonstrated efficacy for PTSD, the strongest evidence exists for trauma-focused interventions, such as cognitive behavioral therapy with a trauma focus ( Department of Veterans Affairs, 2017; International Society for Traumatic Stress Studies, 2019). For these evidence-based treatments, many fail to respond to treatment and dropout rates are high ( Berke *et al*., 2019; Fonzo *et al*., 2020; Kehle-Forbes *et al*., 2016). High dropout rates have been linked to poorer PTSD treatment outcomes ( Hoge *et al*., 2017; Najavits, 2015; Szafranski *et al*., 2014).

Exercise is a subset of physical activity that is planned, structured and repetitive, and has as a final or an intermediate objective the improvement or maintenance of physical fitness ( Caspersen *et al*., 1985). Exercise is a safe, readily accessible, and cost-effective way of improving physical and mental health ( Firth *et al*., 2016).

Exercise has been found to reduce PTSD symptom severity as well as related physical and mental health problems, such as anxiety, depression, pain disorders, sleep disturbances, and cardiovascular diseases ( Oppizzi & Umberger, 2018). Unfortunately, people with PTSD are more likely than the general population to be physically inactive ( Vancampfort *et al*., 2016). As an adjunctive treatment, the potential of exercise has been explored and is promising in individuals with PTSD ( Björkman & Ekblom, 2022; Ramos-Sanchez *et al*., 2021; Stubbs *et al*., 2017; Vancampfort *et al*., 2016; Vancampfort *et al*., 2017). The mechanisms underlying the therapeutic effects of exercise, when incorporated into the treatment of PTSD, are not fully understood ( Crombie *et al*., 2023).

Fear extinction, essential for reducing fear responses to trauma-related cues, is often impaired in individuals with PTSD, resulting in persistent fear and anxiety ( Lokshina *et al*., 2023). These impairments, linked to dysfunctional neural circuits, especially in the amygdala and prefrontal cortex, hinder the retention of extinction memories, sustaining symptoms such as hypervigilance and intrusive thoughts. By helping individuals distinguish between trauma memories and safe present contexts, fear extinction can reduce anxiety and avoidance behaviors in everyday life.

In terms of biomedical mechanisms, it has been hypothesized that exercise-induced changes in neurotransmitters, neurotransmitter modulators, and peptides may modulate molecular processes in key neural pathways underlying enhanced consolidation of extinction learning. Implicated mechanisms include endocannabinoids, dopaminergic signalling, mammalian target of rapamycin (mTOR), and neurotrophic factors such as brain-derived neurotrophic factor (BDNF), which regulates synaptic plasticity, long-term learning, and memory ( Izquierdo *et al*., 2016; Vecchio *et al*., 2018). 

Important to note is the fact that many of the mechanisms that regulate fear extinction are modulated by exercise. This suggests that exercise could be an effective augmentation of psychotherapy for PTSD. Recent investigations suggest that moderate-intensity aerobic exercise during the consolidation window of fear extinction, using exposure-based psychotherapy, reduces threat expectations and symptom severity in women with PTSD ( Crombie *et al*., 2021a; Crombie *et al*., 2021b). For example, previous studies suggest that higher serum levels of BDNF enhance fear extinction. Individuals with low levels of BDNF, like those with Met allele carriers, have impaired fear extinction and thus present with more severe PTSD symptom severity. Exercise modulates genes that regulate BDNF, enhancing fear extinction and reducing PTSD symptom severity ( Pitts *et al*., 2019; Powers *et al*., 2015).

In addition, because of their roles in regulating stress response and fear memories, endocannabinoids are potential target drugs or therapies for PTSD and other trauma-related mental disorders. A case-control study among trauma survivors in Northern Uganda with and without PTSD reported significant reductions in endocannabinoids among survivors with PTSD ( Wilker *et al*., 2016). The authors suggested that the differential reduction in endocannabinoids in survivors with PTSD negatively affected fear extinction. Moreover, another study reported an inverse relationship between the level of endocannabinoids and PTSD symptom severity. PTSD symptom severity could potentially be reduced through exercise-induced elevation of the levels of circulating blood endocannabinoids ( Botsford *et al*., 2023; Crombie *et al*., 2021b). Moderating levels of endocannabinoids is, therefore, a promising strategy for managing PTSD.

Other studies have shown that the effects of anxiolytics and antidepressants likely stem from the effects on neurogenesis through the PGc1alpha-FNDC5-BDNF ( Babaei *et al*., 2021), alterations in kynurenine metabolism ( Notarangelo *et al*., 2018), and normalization of the hypothalamic-pituitary-adrenal axis ( Alghadir & Gabr, 2020). The effects of exercise on enhancing extinction learning and augmenting exposure therapy in PTSD also involves neurochemical events involving the endocannabinoid system ( Marsicano *et al*., 2002), dopaminergic signalling ( Cisler *et al*., 2020; Cui *et al*., 2020; Esser *et al*., 2021), BDNF ( Notaras & van den Buuse, 2020), and mammalian rapamycin ( Moya *et al*., 2020). Further, exercise has been reported to enhance pattern separation and episodic memory ( Déry *et al*., 2013), which is important in differentiating safety from threat and restricting avoidance behaviour. Avoidance behavior, a hallmark symptom of PTSD, reflects an impaired ability to process contextual cues, leading to the generalization of fear responses to non-threatening situations ( Sheynin *et al*., 2017). It has been postulated that this effect is modulated by exercise-induced increases in hippocampal volume ( Erickson *et al*., 2011).

With regard to psychosocial mechanisms, self-efficacy theory, distraction hypothesis, and social interaction have been used to explain the beneficial effects of exercise for PTSD. Self-efficacy refers to an individual’s belief in their ability to meet a desired goal, and has been proposed as a psychological mechanism responsible for the beneficial effects of exercise on mental health ( McAuley & Blissmer, 2000; Robert, 2018). According to the self-efficacy theory, self-efficacy can be increased in several ways; for example, through mastery experiences and vicarious learning ( Bandura, 1977). Negative cognitions/affect, which is a core feature of PTSD, often leads to low self-esteem, self-efficacy and feelings of fear, shame, and guilt ( American Psychiatric Association, 2013; Bonfils *et al*., 2018). Research suggest that exercise can increase self-efficacy and feelings of accomplishment, which can lead to mental health benefits such as decreased anxiety, depression and PTSD symptoms ( Bodin & Martinsen, 2004). The distraction hypothesis proposes that when one is engaged in exercise, it is difficult to focus on their negative thinking and cognitive distortions ( Robert, 2018). Exercise can serve as a distraction from intrusive memories and negative cognitions, core symptoms of PTSD, which can lead to improve mood during and post exercise. Furthermore, exercise has been shown to positively impact resilience and mental health across diverse populations ( Lancaster & Callaghan, 2022; Liu *et al.*, 2024). For example, a cross-sectional study conducted during the COVID-19 pandemic found that exercise, particularly outdoor group activities, enhanced resilience, with stronger effects observed in women ( Lancaster & Callaghan, 2022). These findings highlight the potential of exercise as a valuable strategy for promoting resilience and mitigating the negative effects of traumatic events ( Kochi *et al*., 2017). Lastly, the benefits of social support are well-documented in the PTSD literature. Perceived social support has been associated with reduced PTSD symptom severity, as it is regarded as one of the strongest predictors ( Price *et al*., 2018; Zalta *et al*., 2021). Exercise can serve as a social activity, with mutual support among individuals involved providing a sense of connection with others. This can result in increased social interaction, which in turn can lead to improvements in PTSD related outcomes ( Wilkins *et al*., 2020).

Considering the above, exercise can play an important role in reducing symptom severity and/or improving functional impairment (e.g., disability, quality of life) in patients with PTSD and other trauma-related mental disorders. Functional impairment captures the extent to which symptoms of PTSD interfere with daily activities. This outcome reflects the impact of PTSD on quality of life and day-to-day functioning and is distinct from symptom severity, as a person might experience high functional impairment even with moderate symptom severity, or vice versa ( Ustün & Kennedy, 2009). Nevertheless, the precise mechanisms underpinning the effect of exercise in PTSD management are not fully understood. There is a need to move beyond providing evidence that exercise is effective as an adjunctive treatment, which several studies have already done. Investigation into biological and psychosocial mechanisms of exercise in PTSD and its psychotherapy augmenting mechanisms is an emerging and as yet poorly understood field. Systematic investigation of the underlying mechanisms of its effect and synthesizing these findings into living evidence, which is currently scarce, will be a major contribution to precision care in PTSD ( Crombie *et al*., 2023). It will provide knowledge on the mechanisms of exercise that drive the discovery of other interventions (non-drug and drug) that work through the same or similar mechanisms.

Elucidating the potential mechanisms underlying the beneficial effects of exercise for PTSD is firstly important for fundamental knowledge; secondly, it can shed light on individual-level differences in the effectiveness of exercise for PTSD; and thirdly, it can inform the discovery of other interventions to target these mechanisms. The aim of this living systematic review is to review the evidence on the biopsychosocial mechanisms through which exercise (i.e., aerobic exercise, resistance training, etc.) can facilitate extinction learning, memory, and emotion regulation in PTSD and thereby reduce symptom severity and/or functional impairment.

### Review objectives

To review the biopsychosocial mechanisms through which exercise reduces symptom severity and functional impairment among individuals with PTSD.

### Research questions

***Non-human studies***

I. How does exercise manipulation change stress-related behaviours indicative of animal models of PTSD?
II. What are the effects of exercise on behavioural phenotypes exhibited in animal models of PTSD, and which are the factors that can moderate this?

***Human studies***

I. How does exercise reduce symptom severity and/or functional impairment in PTSD?
II. What are the effects of exercise on symptom severity and functional impairment in individuals with PTSD, and which factors can moderate it?

## Methods for this Living Systematic Review

This living systematic review (LSR) is an output of the Global Alliance for Living Evidence on aNxiety, depressiOn, and pSychosis ( GALENOS) project. A LSR is a systematic review that is continually updated as new research emerges, ensuring the evidence base remains up-to-date. This method is particularly valuable in rapidly advancing fields, where integrating new findings in real-time can significantly improve clinical practice and policy-making ( Elliott *et al*., 2014). The GALENOS project is a global, multidisciplinary consortium aimed at co-producing a continuously updated synthesis of the scientific literature on mental health. The consortium is focused on the underlying mechanisms of depression, anxiety, and psychosis, to inform their prevention and treatment. This protocol is reported in accordance with the PRISMA statement for protocols (PRISMA-P; see Extended Data) ( Shamseer *et al*., 2015; Wright *et al*., 2023). The current study will independently review non-human and human studies.

### Study inclusion and exclusion criteria

The study inclusion and exclusion criteria are provided in Table 1 for the non-human studies and Table 2 for the human studies.

**Table 1.** Study inclusion and exclusion criteria for non-human studies.

*Study design*	*We will include:* Studies will be limited to controlled non-human studies.
*Animal population and* * model induction*	*We will include:* The animal population will include mammals or zebrafish. Zebrafish will be included due to the high genetic homology with mammals, including humans (up to 80%) ( Stewart *et al*., 2015; Yang *et al*., 2020). Performing the same or similar functions, the zebrafish is considered a robust comparative model for PTSD, exhibiting a wide variety of behaviours, complex learning, and neurobiological changes that can be extrapolated to humans. Model induction: We will include (i) studies that claim to model PTSD and (ii) studies that do not make that claim but use the same method of model induction claimed in other studies identified in this review to do so. For PTSD, we will allow pharmacological induction, which is accompanied by a behavioural stressor.
*Experimental interventions/* *exposures*	*We will include:* We will include studies where model animals experience an exercise intervention, whether voluntary or forced. We define exercise as any physical activity or movement of the body through the action of skeletal muscles that would be expected substantially to increase energy expenditure over resting levels. Voluntary exercise is where the animal is presented with the opportunity to engage in exercise but without any “motivating” stimulus (e.g., free access to a running wheel). Forced exercise is where the animal was subjected to a noxious stimulus (e.g., electric shock) if it did not perform the exercise or was restrained such that participation was inevitable.
*Control interventions/non-* *exposures*	*We will include:* We will include experiments where a control group is not exposed to an exercise intervention. Where included experiments also include data from animal cohorts not exposed to stress but exposed to the experimental intervention, these will also be extracted. *We will exclude:* Studies where the level of exercise in the control group is deliberately suppressed below what would be considered normal laboratory animal behaviour.
*Outcomes*	*We will include:* Studies will be included irrespective of the reporting of the outcomes, which are described in the data extraction section. The outcomes of interest are the animal equivalents of PTSD symptom clusters (e.g., freezing behaviour, fear memory, fear generalization, increased startle response, locomotion, and sleep electroencephalography (EEG).

**Table 2.** Study inclusion and exclusion criteria for human studies.

*Study design*	*We will include:* Primary studies with an experimental design in which an exercise intervention is administered. Both randomized and non-randomized controlled trials will be eligible. We will include phase 2 and 3 trials. Studies will be limited to those published in English. Included studies should compare: (i) exercise group versus inactive comparison group (ii) exercise group + psychotherapy versus psychotherapy alone (The psychotherapy aspects will be required to be the same otherwise we will not be able to disentangle the effect of the psychotherapy from the effects of the exercise aspects). *We will exclude in the first version of the review* (see Extended Data for future updates): (i) single arm studies (ii) exercise alone versus psychotherapy alone
*Population*	*We will include:* Participants will be required to have above-threshold symptoms on any standardized self-report measure or a clinical diagnosis of PTSD. No age limit will be placed on study participants.
*Experimental interventions/* *exposures*	*We will include:* The intervention will include (i) exercise alone or (ii) exercise in combination with any psychotherapy for managing PTSD. Exercise will include anaerobic exercises (e.g., resistance and strength training, plyometrics), aerobic exercises, exergaming, or active stretching. The exercise may occur before, during, or after the psychotherapy. The exercise can be of any type, length, and strength; it has to be structured and repetitive to differentiate it from other types of physical activities. *We will exclude:* We will exclude Pilates, martial arts, yoga, tai chi, and horseback riding because of the potential psychological benefits of these activities. Inactive stretching will also be excluded.
*Control interventions/non-* *exposures*	*We will include:* Other comparator interventions such as (i) Wait List Control (WLC), (ii) Treatment as Usual (TAU), or (iii) psychotherapy only groups will be included.
*Outcomes*	Studies will be included irrespective of the reporting or not of the outcomes, which are described in the data extraction section. *Primary outcome:* PTSD total symptom severity *Secondary outcomes:* Symptom cluster severity (avoidance symptoms, reexperiencing symptoms, hyperarousal symptoms, and negative cognitions and mood) Functional impairment Loss of PTSD diagnosis post-intervention Dropout

### Study identification

The search strategy will be defined in collaboration with the search team. The ontology team will be informed of the search strategy and will help identify additional search terms where possible and relevant. The resulting search strategy will also inform the scope of the ontology. An ontology protocol will be available and will be included as Extended Data. See Extended Data for the Galenos mental health ontology development overview.

***Non-human studies***

In this first iteration, we will use a conventional search strategy (Title [Ti], Abstract [Ab], Keywords and Medical Subject Headings [MeSH] over PubMed (including pre-prints), Web of Science, Scopus and PsycInfo. MeSH is a comprehensive controlled vocabulary used by the National Library of Medicine to index articles for PubMed, enabling precise and consistent retrieval of information on specific medical topics. We will also search for unpublished preclinical and animal studies in registries (e.g., animalstudyregistry.org, preclinicaltrials.eu) and preprint databases (e.g., medRxiv, bioRxiv).

***Human studies***

In this first iteration, we will use a conventional search strategy (Ti/Ab/Keyword/MeSH). The electronic searches will include major literature databases such as, MEDLINE via Pubmed (including preprints), Embase, Scopus, Web of Science, PTSDpubs, Biosis, CINAHL (nursing and allied health), PsycInfo, SportDiscus, and Cochrane Library from inception. We will search for unpublished human studies in clinical trial registries (e.g., ClinicalTrials.gov, WHO-ICTRP). Reference lists of included studies, relevant reviews ( Björkman & Ekblom, 2022; Crombie *et al*., 2023; Hegberg *et al*., 2019), and conference proceedings will also be screened for additional articles yet to be identified. The search strings will combine terms related to exercise, trauma, stress disorders, and posttraumatic stress. The searches will combine free and indexed terms. Boolean operators will also be utilized. The full search strategy will be added as Extended Data (see Extended Data for the MEDLINE search strategy).

### Study selection

This review will be reported in line with the Preferred Reporting Items for Systematic Reviews and Meta-Analyses (PRISMA) Statement ( Page *et al*., 2021). We will record the selection process in sufficient detail during the study selection to complete a PRISMA flow diagram and 'Characteristics of excluded studies' table. A table of excluded studies which will refer to studies meeting the inclusion criteria but failed in one or more exclusion criteria, will be provided as Extended Data.

***Non-human studies***

Search results will be de-duplicated using the Automated Systematic Search Deduplicator (ASySD) ( Hair *et al*., 2021) and then uploaded to the Systematic Review Facility (SyRF)( Bahor *et al*., 2021). After electronic deduplication of the search results, the study selection will be conducted in two levels (e.g., title/abstract and full text). Each record will undergo at least two independent screening decisions (‘include’ or ‘exclude’), and in the case of disagreement, the record will automatically be offered to a third independent reviewer. We will retrieve the full texts of “relevant” and “unclear” records from the title/abstract screening, which will be screened by at least two independent reviewers for eligibility. In the case of disagreement, the record will automatically be offered to a third independent reviewer. If the full text is still unclear or there is no available full publication of the record, the authors will be contacted to provide further information.

***Human studies***

The results of our academic searches will be imported into Evidence for Policy & Practice Information Centre reviewer (EPPI) ( EPPI reviewer, 2023). Twenty percent of titles and abstracts of all the identified studies will be independently examined by two members of the review team and deemed eligible or ineligible. One reviewer will screen the remaining abstracts; a second will screen all excluded abstracts, resolving any uncertainties regarding study inclusion.

For full-text screening, we will use a standardized form and conduct a pilot exercise using the same 5–10 articles for the entire screening team to test the review form. Two reviewers will examine 20% of the eligible full texts. One reviewer will screen all included full texts, while the second will screen all excluded ones. A third review team member will resolve any disagreements regarding study inclusion. When no full publication of an eligible abstract can be located, the authors will be contacted to provide further information.

### Outcomes and prioritization

***Non-human studies***

The outcome data that will be included are the animal equivalents of PTSD symptom clusters. For example, low/high levels of freezing behaviour, fear memory (e.g., recognition memory tasks), fear generalization, increased or decreased startle response, locomotion (e.g., Social Interaction Test) ( File & Seth, 2003), arousal (e.g., Social Interaction Test) ( File & Seth, 2003), and sleep EEG (e.g., increased or decreased awakenings from rapid eye movement (REM), increased or decreased duration of REM, recurrent nightmares).

***Human studies***

The primary outcome will be PTSD total symptom score as an indicator of symptom severity. Secondary outcomes are avoidance symptom severity, reexperiencing symptom severity, hyperarousal symptom severity, negative cognitions and mood severity, functional impairment (e.g., disability, quality of life), loss of PTSD diagnosis post-intervention, and dropout rates. PTSD total and cluster symptom severity scores will be measured using validated PTSD self-report measures such as the Impact of Event Scale-Revised (IES-R) ( Weiss & Marmar, 1997) or clinical interviews such as the Clinician-Administered PTSD Scale (CAPS-5) ( Weathers *et al*., 2013). Functional impairment will be measured using validated measurement scales, such as the Sheehan Disability Scale (SDS) ( Sheehan & Sheehan, 2008) We will accept all relevant self-reported or observer-measured measures that assess these constructs. Any adverse effects reported in the primary studies will be extracted.

### Data extraction

Data extraction will be conducted in SyRF ( Bahor *et al*., 2021) for non-human studies and EPPI ( EPPI reviewer, 2023) for human studies. The data extraction form will be sent to the ontology team to identify relevant ontology categorizations to support data extraction. The data extraction forms (and annotations for non-human studies) will be tested to confirm that all the relevant data will be captured. Revisions will be made, if judged appropriate before we embark on the full data extraction. Two independent reviewers will perform annotation and data extraction using the piloted data extraction forms. Any differences in extracted data between the reviewers will be resolved through discussion with a third reviewer on the team.

***Examining the associations between exercise, mediators and outcomes***

**Figure 1a.**
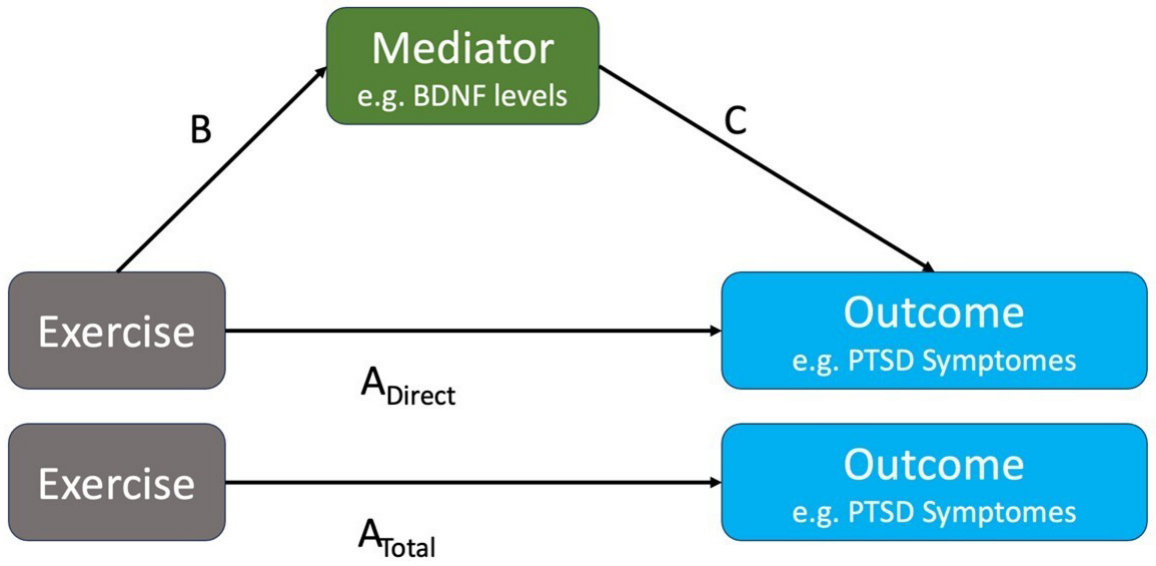
Example of a mediation of the effect of exercise on improving PTSD symptoms via changing the levels of BDNF. Assuming a continuous outcome and mediator, the indirect effect of exercise on the outcome A _Indirect_ is obtained as the product of B and C effect sizes. The direct effect A _Direct_ would typically not be reported in studies; instead, the total effect A _Total_= A _Direct_+A _Indirect_ is reported.

To explain the biopsychosocial mechanisms through which exercise affects the outcome of interest (PTSD symptom severity) for research questions I in humans and animals, we will examine potential mediating variables. A mediating variable directly affects the relationship between the independent and dependent variables, which provides an (often partial) explanation for why the relationship exists. To give a biomedical example, it has been hypothesized that exercise (independent variable) modulates genes that regulate BDNF (mediator), which in turn reduces PTSD symptom severity (dependent variable/outcome). Similarly, in terms of psychosocial mechanisms, exercise (independent variable) can lead to increased self-efficacy (mediator), which in turn reduces PTSD symptom severity (dependent variable/outcome). Figure 1a shows a very simplified version of the mediation of the effect of exercise on PTSD symptom severity mediated via BDNF. For research questions I, we will extract data that evaluate the associations of type B (the impact of exercise on a mediator) and C (the impact of the mediator on the outcome).

To gain a deeper understanding of research question II in human studies, we will extract data on impact of exercise on A _Total_ for PTSD symptom severity and functional impairment, and we will examine potential effect modifiers using standard meta-analytic methods. Effect modifiers affect the strength of the relationship between exercise and PTSD symptoms, and by examining them, we might gain some insight into what mechanisms are at play. For example, if sex was found to be a strong modifier of the relationship between exercise and PTSD symptom severity, this could be indicative of different mechanisms of action between males and females.

The potential mediators and effect modifiers are currently unknown so, for both non-human and human studies, we make some assumptions below about the variables we suspect could be either mediators or modifiers. Data will be extracted for any variable the authors report as being a moderator or mediator of the association between exercise and the outcome variable of interest. 

***Extracting numerical data for the associations between exercise, mediators, and outcomes***

We expect that many of the associations A, B, and C in Figure 1a will be only descriptive, and we will extract statements about associations as reported.

*For associations between a dichotomous variable and a continuous* (e.g., between exercise and PTSD symptoms), we will extract the mean, standard deviation (SD), and sample size for each group. If these are not reported, we will calculate them from standard error of the mean, p-values, t-tests, F-tests, or confidence intervals. When only median/ranges are reported, we will extract them.

*For associations between two dichotomous variables (e.g., exercise and high/low BDNF levels)*, we will extract the numbers from 2x2 cross-classified tables.

*For associations between two continuous variables (only possible in associations C, e.g., BDNF levels and PTSD symptoms),* we will extract the mean difference in the outcome for one level change in the assumed mediator, ideally obtained from regression analysis (i.e., the coefficient of regression and its SE).

If proper mediation analysis has been performed in the study, we will record this and extract data about all potential mediators accounted for.

We shall attempt to minimize missing outcome or mediator data through (i) the inclusion of results from trial registries; (ii) the inclusion of results from clinical study reports and other regulatory documents; and (iii) contacting the authors. We will extract data from methods that account for missing data, giving preferences to (i) mixed-models of repeated measurement (MMRM) and multiple imputations, followed by (ii) last-observation carried forward (LOCF), and finally (iii) observed cases.

Dropouts for any reason will be conceptualized as any participant who failed to complete the post-intervention assessment in accordance with prior meta-analytical research in the field of PTSD ( Lewis *et al*., 2020). When possible, we will extract the timing of the dropout data. Missing outcome data will be considered in the risk of bias assessments (see *Risk of bias assessment*). In crossover trials, data will be extracted for the first phase of the study (before crossover) to avoid carryover effects.

***Non-human studies***

The primary meta-analysis of interest for animal studies is of data from experiments where the Single Prolonged Stress model of PTSD has been used ( Liberzon *et al*., 1997). Several other animal models which recapitulate features of PTSD have been described many of which have a primary or auxiliary application in modelling other human conditions ( Freund & Juckel, 2019). In later iterations of this review, we will extend our analysis to include these other models, including all such studies and not limited to those where the authors have specifically set out to model PTSD.

When the sample size is not adequately reported, it will be estimated when possible (e.g., using the low boundary of a range) ( Vesterinen *et al*., 2014). There are often relatively trivial differences in values extracted from graphs for continuous data, therefore we will flag for reconciliation those data points for which the discrepancy between the two reviewers is greater than 10%. For differences less than 10%, the mean of the two values will be taken.

Several measurement tools or variations for the same tool are likely used to measure the same outcome. We will extract data from all reported variations and any available correlation/covariance in such cases. These data will be jointly synthesized. When an intervention is provided as several treatments over an extended period, data from the last time point in the treatment period will form the primary analysis. When the intervention is conducted within a 24-hour period, and a monophasic response is expected, we will calculate the area under the curve and its variance.

*Potential mediators*

We will extract data relevant to the potential mechanisms of any effect of exercise on changes in the behavioural manifestations of the modelling of PTSD symptoms. Biomedical mediators include but will not be limited to (i) changes in synaptic function or plasticity observed in *in vivo* or *ex vivo* electrophysiology; and (ii) changes in the abundance (CSF or plasma levels, immunohistochemistry, quantitative western blot) of proteins or expression (mRNA levels, in situ hybridization) of genes such as BDNF which may be involved in mediating any effect. External mediators include but are not limited to, the stimulation of moving location as a result of exercise.

*Potential effect modifiers*

Potential effect moderators will be extracted, such as study characteristics (e.g., species, strain, sex, age, weight, comorbidity), exercise characteristics (e.g., exercise type – voluntary or forced, duration, intensity, control conditions), and outcome characteristics such as measurement parameters used (e.g., fear-related behaviour, fear conditioning).

***Human studies***

When available, we will extract follow-up data at the following time points: (i) pre-intervention (baseline), (ii) primary endpoint is the first post-intervention time point reported or the end of the study (if exercise is given for the entire study duration) (iii) all post-intervention follow-up assessments.

*Potential mediators*

Potential mediators will be extracted as putative explanatory mechanisms for the effects of exercise on the primary outcome (PTSD symptom severity), such as fear potential startle (e.g., electrodermal activity, pulse and heart rate, eyeblink rate), saliva and blood analyses (e.g., BDNF, eCB ligands [e.g., anandamide, 2-arachidonoylglycerol, N-acyl ethanolamine (NAE) molecules such as palmitoylethanolamide and oleoylethanolamide]), neuroendocrine assays, inflammatory markers (e.g., cytokines such as IL-6, TNF-α), neuroimaging markers (e.g., MRI brain morphometry and function), self-esteem, self-efficacy, social interaction, social support, recall tests, and threat expectancy ratings.

*Potential effect modifiers*

Potential moderators will be extracted, such as study characteristics (e.g., design), control intervention, participants characteristics (population, sex, age, weight, comorbidities). Intervention characteristics will include exercise frequency (number of intervention sessions per week), intervention length (total number of sessions intervention conducted over), duration of exercise session (planned, and average length in minutes), exercise sequencing (time of administration relative to safety learning: before, after, or during psychotherapy/extinction learning), exercise type (e.g., anaerobic: strength / resistance training; aerobic/endurance – running, jogging, cycling etc; explosive/ power training – sprinting, box jumps; active/ dynamic stretching; mixed; other ), exercise intensity (high vs. low intensity; vo2 max), intervention format (group vs. individual vs. mixed), and intervention setting (community vs. primary care vs. secondary/tertiary care), timing of the primary timepoint (see below), genetics (e.g., candidate genes such as the BDNF Val66Met polymorphism), gene expression (e.g. differential BDNF gene expression), epigenetics (e.g., differential BDNF methylation), patient expectancy of outcome (e.g., Credibility and Expectancy Questionnaire)( Devilly & Borkovec, 2000), baseline clinical comorbidities (e.g., depression, anxiety, and substance use), and therapist expectancy of outcome (e.g., Therapist Expectancy Inventory)( Bernstein *et al*., 1983).

### Risk of bias assessment

The risk of bias will be assessed for the primary outcomes of animal and human studies. We will evaluate the risk of bias for animal studies using the SYRCLE tool ( Hooijmans *et al*., 2014). The ROBINS-I ( Sterne *et al*., 2016) will be used for non-randomized human trials, and Cochrane Risk of Bias 2 (RoB2) ( Higgins *et al*., 2022) for randomized human trials. For non-animal studies, the completeness of reporting of study design, conduct, and analysis is a pre-requisite for evaluation of risks of bias. To evaluate whether the measurement of the mediators have been done in an appropriate manner, we will extract the timing of the mediator assessment (before or at the same time as the outcome), and whether confounders between the mediators and the outcome have been considered.

Reporting is often incomplete, meaning that many publications are graded as having ‘unclear’ risk of bias across multiple dimensions.

A single reviewer will rate the risk of bias for the primary outcome for each included study, with a full verification of all judgments by a second reviewer. A third reviewer will resolve any uncertainties. For each tool, the overall risk of bias for each study, on each primary outcome, will be summarised as follows: (i) at high risk of bias: when at least one of the domains was judged at high risk of bias or above (ii) at low risk of bias: when at most one of the domains was judged at moderate risk of bias (iii) Some concerns about bias: all other cases. The impact of risk of bias will be assessed by sensitivity analysis by restricting studies to those with an overall low risk of bias.

### Data analysis and synthesis

***Effect sizes for the recorded associations***

Using the data extracted for each association A _Total_, B, and C in Figure 1, we will calculate effect sizes according to the nature of the data.

For continuous data, the effect size will be the mean difference (MD) if all studies use the same scale or unit of measurement; otherwise, we will calculate the standardized mean difference (SMD). We will ensure the direction of the effect is the same when outcomes are measured on different scales (e.g., for PTSD symptom severity, a higher score indicating more severe PTSD symptom severity). For non-human experiments, we will consider a sensitivity analysis using normalized mean differences (NMD) when outcomes are measured on different scales/units, and the performance of untreated animals can be known or inferred in the majority of the studies. Dichotomous data will be summarized as risk ratio (RR).

***Data synthesis***

*Research questions I: Exploring the role of mediators*

Comparison of within-study direct and indirect effects A _ind_ and A _direct_ obtained from mediation analysis gives insight into the importance of the examined mediator. We will collect all study-specific estimates of the effects of type B, C, A _ind_ and A _direct_ and will group them by mediator. As B and C might not have been calculated from proper mediation analyses, A _ind_ and A _direct_ would be only crude estimates of the true direct and indirect effects of exercise on the outcome. If enough estimates of sufficient quality are extracted for the same mediator, these can be synthesized across studies using random-effects meta-analyses ( Cheung, 2022).

*Research question II: The impact of exercise on PTSD symptoms and function impairment*

We expect several estimates of type A _total_ to be available for potential synthesis from human studies. Heterogeneity will be assessed by visually inspecting forest plots considering the direction and magnitude of effects and the degree of overlap between Cis. If the heterogeneity is not judged to be substantial, we will conduct random-effects meta-analysis using the restricted maximum likelihood estimator of the between-study variance (τ ^2^). We will the Hartung-Knapp correction for the confidence intervals of the summary estimate when at least five studies are available. Heterogeneity will be quantified using the τ ^2 ^and presented with the 95% prediction intervals (95% PI) of the treatment effects. When a meta-analysis is not feasible, we will synthesize the data without an accompanying meta-analysis (SwiM) ( Campbell *et al*., 2020). Furthermore, for non-human studies, we will conduct multilevel multivariable meta-regression with robust variance estimation (RVE).

When a meta-analysis is possible, and there is sufficient data, we will examine potential characteristics as sources of heterogeneity by conducting subgroup analysis and meta-regression for the primary outcome. The potential effect modifiers are listed in the data extraction section above. We will also perform subgroup analysis for study design characteristics such as type of comparator.

***Sensitivity analyses***

When there is sufficient data for a meta-analysis, we will conduct a sensitivity analysis to determine the effect of limiting the analysis to only studies rated with a low risk of bias on the primary outcome.

### Reporting bias

We will consider both within-study (selective outcome reporting in a study) and across-study (studies undertaken but not reported) assessments of reporting bias and assess the potential effect on the magnitude/direction of the findings. We will examine the basis for selective non-reporting of results.

Publication bias will be examined considering the direction and magnitude of the effects. For non-human studies, when there are 10 or more studies included, we will use multivariate versions of Egger’s regression test to assess for small study effects. For human studies, when there are 10 or more studies included, we will inspect the contour-enhanced funnel plots and use Egger’s test of funnel plot asymmetry for continuous outcomes ( Egger *et al*., 1997) and Peters test for dichotomous outcomes ( Peters *et al*., 2008) to assess for small-study effects. 

We will evaluate the summary of the evidence using an adapted version of the GRADE framework ( Schünemann *et al*., 2013).

### Summary of the evidence

We will evaluate the Summary of Evidence (SoE) tables for non-human and human studies separately.

For research questions I, a SoE on mediators will provide:

(i) A list of variables that have been examined as mediators in the included studies;

(ii) Characteristics of the evidence about each candidate mediator including the number of studies that examine it, estimated direct and indirect effects or descriptive statements about the mediation potential from study authors, and the credibility of the studies concerning the proper examination of the mediation.

Credibility of the studies will include descriptive information on the risk of bias on the studies that report the mediator, the appropriateness of timing in the measurement of the mediator in the included studies, and the level of adjustment in the effect of the mediator on the outcome in the included studies. We will try to find a correspondence of the outcomes and mediators examined and human and non-human studies, when possible, with input from the ontology group.

For research questions II, we will consider: (i) source of evidence; (ii) summary of associations; (iii) bias due to study limitations; (iv) bias due to reporting bias; (v) bias due to indirectness; (vi) bias due to other reasons. Table 3 presents the structure of the SoE table and the domains that will be completed for the primary outcome (PTSD symptom severity).

**Table 3.** Summary of evidence table.

Source of evidence	Summary of the association	Bias due to study limitations	Bias due to reporting bias	Bias due to indirectness: *population*	Bias due to other reasons
Systematic review of human studies (potentially further grouped by design, e.g., phase I/II separated from phase III)	Numerical summary from the meta-analysis (point estimate, confidence interval, and prediction interval), Numerical SWiM range, or qualitative statement about the association (and its direction).	State the risk of bias in the summary conclusions due to within-study limitations (low, moderate, high) and the expected direction of bias (over or underestimating the true effect). If needed, separate this column in several domains e.g., missing outcome data, blinding, etc. This might help you with guessing the direction of bias.	State the risk of bias in the summary conclusions due to reporting bias limitations (low, moderate, high) and the expected direction of bias.	State the risk of bias due to populations included not relevant to the target population. Make a statement about the expected direction of bias (over or underestimation of the true effect).	Discuss here any other biases that are not covered.

### Consensus meeting to select possible mediators

Human and non-human studies produce different types of evidence on the mechanisms through which exercise can facilitate extinction learning, memory, and emotion regulation in PTSD. However, non-human studies have provided us with a deeper understanding of the mechanisms behind human functioning, leading to substantial advancements in the medical field ( Giannakou & Vyrides, 2021).

The evidence about the potential mediators of the association between exercise and PTSD-related outcomes from human and non-human studies will be considered in an expert meeting when there is sufficient evidence available from at least two sources. The participants in the meeting will consider the evidence from the human and non-human studies as summarized in the Summary of the Evidence on Moderators (SEM). We will attempt to organize the examined mediators according to a hierarchy of association and classify them as probable or improbable mediators of the association. We will also consider whether the review should continue for some or all studied outcomes, and the type of update that should be done (see SUMMARY OF THE EVIDENCE, for research questions I).

### Updating the systematic review and stopping the living mode of the review

This study will be set up as an LSR, a high-quality online summary of the scientific literature updated as new research that meets study inclusion criteria becomes available ( Elliott *et al*., 2014). Once we have completed the first version of the systematic review, the methods applied will be reconsidered to judge their suitability and efficiency in practice. If judged to be appropriate, the methodological approaches of data extraction and synthesis will be amended accordingly, and any changes will be documented. The literature search will be updated every three months, and any new studies meeting the inclusion criteria will be incorporated into the latest version of the evidence. If sufficient new data exists, a triangulation meeting will be held to incorporate the new evidence. Furthermore, it will be decided whether the review updates will continue, and whether to widen the scope of the review (this may include contacting authors of major studies to provide missing data). When there is insufficient new data to warrant triangulation, the review will be updated again after three months. We will also consider whether to incorporate hypothesis testing of identified probable mediators as a specific aim going forward. The living systematic review will use a versioning system based on the one used by F1000. We will clearly report all updates, and any deviations from the methods outlined in this protocol will be documented and justified.

### Co-production aspects

We have employed a multidisciplinary approach by considering the perspectives, experience, and knowledge of multiple stakeholders such as preclinical and clinical researchers, clinicians, systematic review methodologists, statisticians, and experiential advisors.

In formulating the focus of the review, we utilized existing prioritization exercises that had co-production embedded in their process, namely, the UK MH research goals 2020–2023, the WHO Grand Challenges in Mental Health ( Collins *et al*., 2011) and the James Lind Alliance’s Top 10 Priorities for Depression (2016) and Schizophrenia (2011). Common themes emerged, including research to develop new and improved treatments, root causes, a better understanding of therapeutic mechanisms for current drug and psychological treatments, and this is the starting point for the initial LSR questions within GALENOS.

To ensure the comprehensive consideration of perspectives from all stakeholders involved, we will assemble a team of co-authors who represent the diverse backgrounds mentioned above. It is anticipated that each co- author will substantially contribute more to specific sections based on their individual experiences and expertise. The review team will be provided with guidance by work package 1 (WP1) on effective models of involvement for Experiential Advisors. As a result, a multidisciplinary approach will be implemented throughout all stages of the review, from identifying needs, formulating the research aims, designing the review, and interpreting and disseminating the findings to the research and public community.

Considering the complexity and multidimensionality of the review topic, we will establish a schedule of regular team meetings and foster effective communication within the GALENOS project. The primary objective of these initiatives is to facilitate a shared understanding, promote the transferability of knowledge, encourage the exchange of ideas and perspectives, and identify the distinct needs of various stakeholders. By implementing these measures, we aim to create an environment where all stakeholders have equal standing and can actively contribute to the collaborative production of the review.

### Dissemination of information

This LSR will be published via the GALENOS Gateway on Wellcome Open Research, the GALENOS website, and the quarterly Research Roundup newsletter on Mental Health Research issues. A Plain English summary will accompany the review. We will use social media (e.g., Twitter, Facebook) and write blog posts that will be available on the GALENOS website to disseminate our findings further. Furthermore, we aim to present GALENOS at the World Congress of Biological Psychiatry as well as other conferences.

### Study status

The study status at the date of submission 11.08.2023 is reported below.

***Preliminary searches***

Started but not completed.

***Piloting the study selection process***

Not started.

***Piloting the study selection process***

Not started.

***Full searches***

Not started.

***Full screening of search results against eligibility criteria***

Not started.

***Data extraction***

Not started.

***Risk of bias or quality assessment***

Not started.

***Data synthesis***

Not started.

## Data Availability

No data are associated with this article. Open Science Framework: Extended data for ‘Mechanisms through which exercise reduces symptom severity and/or functional impairment in posttraumatic stress disorder (PTSD): Protocol for a living systematic review of human and non-human studies’, https://doi.org/10.17605/OSF.IO/GXWJN ( Wright *et al*., 2023) This project includes the following extended data: Future updates.docx Search strategy for MEDLINE.docx (Open Science Framework: GALENOS, https://doi.org/10.17605/OSF.IO/WMGDQ (also CC-BY 4.0)) Open Science Framework: PRISMA-P checklist for ‘Mechanisms through which exercise reduces symptom severity and/or functional impairment in posttraumatic stress disorder (PTSD): Protocol for a living systematic review of human and non-human studies’, https://doi.org/10.17605/OSF.IO/GXWJN ( Wright *et al*., 2023) Data are available under the terms of the Creative Commons Attribution 4.0 International license (CC-BY 4.0)

## References

[ref-1] Alghadir AH Gabr SA : Hormonal function responses to moderate aerobic exercise in older adults with depression. *Clin Interv Aging.* 2020;15:1271–1283. 10.2147/CIA.S259422 32821089 PMC7423410

[ref-2] American Psychiatric Association: Diagnostic and Statistical Manual of mental disorders (5th ed.). American Psychiatric Publishing,2013. Reference Source

[ref-3] Babaei A Nourshahi M Fani M : The effectiveness of continuous and interval exercise preconditioning against chronic unpredictable stress: involvement of hippocampal PGC-1α/FNDC5/BDNF pathway. *J Psychiatr Res.* 2021;136:173–183. 10.1016/j.jpsychires.2021.02.006 33607579

[ref-4] Bahor Z Liao J Currie G : Development and uptake of an online systematic review platform: the early years of the CAMARADES Systematic Review Facility (SyRF). *BMJ Open Sci.* 2021;5(1): e100103. 10.1136/bmjos-2020-100103 35047698 PMC8647599

[ref-5] Bandura A : Self-efficacy: toward a unifying theory of behavioral change. *Psychol Rev.* 1977;84(2):191–215. 10.1037//0033-295x.84.2.191 847061

[ref-6] Berke DS Kline NK Wachen JS : Predictors of attendance and dropout in three Randomized Controlled Trials of PTSD treatment for active duty service members. *Behav Res Ther.* 2019;118:7–17. 10.1016/j.brat.2019.03.003 30933748

[ref-7] Bernstein BL Lecomte C Des Harnais G : Therapist expectancy inventory: development and preliminary validation. *Psychol Rep.* 1983;52(2):479–487. 10.2466/pr0.1983.52.2.479 6878542

[ref-8] Björkman F Ekblom Ö : Physical exercise as treatment for PTSD: a systematic review and meta-analysis. *Mil Med.* 2022;187(9–10):e1103–e1113. 10.1093/milmed/usab497 34850063

[ref-9] Bodin T Martinsen EW : Mood and self-efficacy during acute exercise in clinical depression. A randomized, controlled study. *J Sport Exerc Psychol.* 2004;26(4):623–633. 10.1123/jsep.26.4.623

[ref-10] Bonfils KA Lysaker PH Yanos PT : Self-stigma in PTSD: prevalence and correlates. *Psychiatry Res.* 2018;265:7–12. 10.1016/j.psychres.2018.04.004 29679793

[ref-11] Botsford C Brellenthin AG Cisler JM : Circulating endocannabinoids and psychological outcomes in women with PTSD. *J Anxiety Disord.* 2023;93: 102656. 10.1016/j.janxdis.2022.102656 36469982 PMC9839585

[ref-12] Bromet EJ Atwoli L Kawakami N : Post-Traumatic Stress Disorder associated with natural and human-made disasters in the world mental health Surveys. *Psychol Med.* 2017;47(2):227–241. 10.1017/S0033291716002026 27573281 PMC5432967

[ref-13] Campbell M McKenzie JE Sowden A : Synthesis Without Meta-analysis (SWiM) in systematic reviews: reporting guideline. *BMJ.* 2020;368: l6890. 10.1136/bmj.l6890 31948937 PMC7190266

[ref-14] Caspersen CJ Powell KE Christenson GM : Physical activity, exercise, and physical fitness: definitions and distinctions for health-related research. *Public Health Rep.* 1985;100(2):126–131. 3920711 PMC1424733

[ref-15] Cheung MW : Synthesizing indirect effects in mediation models with meta-analytic methods. *Alcohol Alcohol.* 2022;57(1):5–15. 10.1093/alcalc/agab044 34190317

[ref-16] Cisler JM Privratsky AA Sartin-Tarm A : L-DOPA and consolidation of fear extinction learning among women with Posttraumatic Stress Disorder. *Transl Psychiatry.* 2020;10(1): 287. 10.1038/s41398-020-00975-3 32801342 PMC7429959

[ref-17] Collins PY Patel V Joestl SS : Grand challenges in global mental health. *Nature.* 2011;475(7354):27–30. 10.1038/475027a 21734685 PMC3173804

[ref-18] Crombie KM Adams TG Dunsmoor JE : Aerobic exercise in the treatment of PTSD: an examination of preclinical and clinical laboratory findings, potential mechanisms, clinical implications, and future directions. *J Anxiety Disord.* 2023;94: 102680. 10.1016/j.janxdis.2023.102680 36773486 PMC10084922

[ref-19] Crombie KM Sartin-Tarm A Sellnow K : Aerobic exercise and consolidation of fear extinction learning among women with posttraumatic stress disorder. *Behav Res Ther.* 2021a;142: 103867. 10.1016/j.brat.2021.103867 34020153

[ref-20] Crombie KM Sartin-Tarm A Sellnow K : Exercise-induced increases in Anandamide and BDNF during extinction consolidation contribute to reduced threat following reinstatement: preliminary evidence from a Randomized Controlled Trial. *Psychoneuroendocrinology.* 2021b;132: 105355. 10.1016/j.psyneuen.2021.105355 34280820 PMC8487992

[ref-21] Cui W Aida T Ito H : Dopaminergic signaling in the Nucleus Accumbens modulates stress-coping strategies during inescapable stress. *J Neurosci.* 2020;40(38):7241–7254. 10.1523/JNEUROSCI.0444-20.2020 32847967 PMC7534921

[ref-22] Department of Veterans Affairs: VA/DoDClinical Practice Guideline for the Management of Posttraumatic Stress Disorder and Acute Stress Disorder.2017. Reference Source

[ref-23] Déry N Pilgrim M Gibala M : Adult hippocampal neurogenesis reduces memory interference in humans: opposing effects of aerobic exercise and depression. *Front Neurosci.* 2013;7:66. 10.3389/fnins.2013.00066 23641193 PMC3639381

[ref-24] Devilly GJ Borkovec TD : Psychometric properties of the credibility/expectancy questionnaire. *J Behav Ther Exp Psychiatry.* 2000;31(2):73–86. 10.1016/s0005-7916(00)00012-4 11132119

[ref-25] Egger M Davey Smith G Schneider M : Bias in meta-analysis detected by a simple, graphical test. *BMJ.* 1997;315(7109):629–634. 10.1136/bmj.315.7109.629 9310563 PMC2127453

[ref-26] Elliott JH Turner T Clavisi O : Living systematic reviews: an emerging opportunity to narrow the evidence-practice gap. *PLoS Med.* 2014;11(2): e1001603. 10.1371/journal.pmed.1001603 24558353 PMC3928029

[ref-27] EPPI reviewer.2023. Reference Source

[ref-28] Erickson KI Voss MW Prakash RS : Exercise training increases size of hippocampus and improves memory. *Proc Natl Acad Sci U S A.* 2011;108(7):3017–3022. 10.1073/pnas.1015950108 21282661 PMC3041121

[ref-29] Esser R Korn CW Ganzer F : L-DOPA modulates activity in the vmPFC, nucleus accumbens, and VTA during threat extinction learning in humans. *eLife.* 2021;10: e65280. 10.7554/eLife.65280 34473055 PMC8443250

[ref-30] File SE Seth P : A review of 25 years of the social interaction test. *Eur J Pharmacol.* 2003;463(1–3):35–53. 10.1016/s0014-2999(03)01273-1 12600701

[ref-31] Firth J Rosenbaum S Stubbs B : Motivating factors and barriers towards exercise in severe mental illness: a systematic review and meta-analysis. *Psychol Med.* 2016;46(14):2869–2881. 10.1017/S0033291716001732 27502153 PMC5080671

[ref-32] Fonzo GA Federchenco V Lara A : Predicting and managing treatment non-response in Posttraumatic Stress Disorder. *Curr Treat Options Psychiatry.* 2020;7(2):70–87. 10.1007/s40501-020-00203-1 33344106 PMC7748158

[ref-33] Freund N Juckel G : Bipolar disorder: its etiology and how to model in rodents. *Methods Mol Biol.* 2019;2011:61–77. 10.1007/978-1-4939-9554-7_4 31273693

[ref-34] Giannakou K Vyrides A : The use of animal studies in human research. *Archives of Hellenic Medicine.* 2021;38(6):761–765. Reference Source

[ref-35] Goldstein RB Smith SM Chou SP : The epidemiology of DSM-5 Posttraumatic Stress Disorder in the United States: results from the national epidemiologic survey on alcohol and related conditions-III. *Soc Psychiatry Psychiatr Epidemiol.* 2016;51(8):1137–1148. 10.1007/s00127-016-1208-5 27106853 PMC4980174

[ref-36] Hair K Bahor Z Macleod M : The Automated Systematic Search Deduplicator (ASySD): a rapid, open-source, interoperable tool to remove duplicate citations in biomedical systematic reviews. *bioRxiv.* 2021; 2021.2005.2004.442412. 10.1101/2021.05.04.442412 PMC10483700 37674179

[ref-37] Hegberg NJ Hayes JP Hayes SM : Exercise intervention in PTSD: a narrative review and rationale for implementation. *Front Psychiatry.* 2019;10: 133. 10.3389/fpsyt.2019.00133 30949075 PMC6437073

[ref-38] Higgins JPT Savović J Page MJ : Cochrane handbook for systematic reviews of interventions version.In: J. P. T. Higgins, J. Thomas, J. Chandler, M. Cumpston, T. Li, M. J. Page, & V. A. Welch (Eds.), *Assessing risk of bias in a randomized trial. *Cochrane Training,2022.

[ref-39] Hoge CW Lee DJ Castro CA : Refining Trauma-Focused Treatments for servicemembers and veterans with Posttraumatic Stress Disorder: progress and ongoing challenges. *JAMA Psychiatry.* 2017;74(1):13–14. 10.1001/jamapsychiatry.2016.2740 27893037

[ref-40] Hooijmans CR Rovers MM de Vries RB : SYRCLE’s risk of bias tool for animal studies. *BMC Med Res Methodol.* 2014;14(1): 43. 10.1186/1471-2288-14-43 24667063 PMC4230647

[ref-41] International Society for Traumatic Stress Studies: ISTSS guidelines position paper on complex PTSD in adults. 2019. Reference Source

[ref-42] Izquierdo I Furini CR Myskiw JC : Fear memory. *Physiol Rev.* 2016;96(2):695–750. 10.1152/physrev.00018.2015 26983799

[ref-43] Kehle-Forbes SM Meis LA Spoont MR : Treatment initiation and dropout from prolonged exposure and cognitive processing therapy in a VA outpatient clinic. *Psychol Trauma.* 2016;8(1):107–114. 10.1037/tra0000065 26121175 PMC9873270

[ref-91] Kochi C Liu H Zaidi S : Prior treadmill exercise promotes resilience to vicarious trauma in rats. *Prog Neuropsychopharmacol Biol Psychiatry.* 2017;77:216–221. 10.1016/j.pnpbp.2017.04.018 28428145 PMC5613975

[ref-92] Lancaster MR Callaghan P : The effect of exercise on resilience, its mediators and moderators, in a general population during the UK COVID-19 pandemic in 2020: a cross-sectional online study. *BMC Public Health.* 2022;22(1): 827. 10.1186/s12889-022-13070-7 35468747 PMC9037056

[ref-44] Lewis C Roberts NP Andrew M : Psychological therapies for Post-Traumatic Stress Disorder in adults: systematic review and meta-analysis. *Eur J Psychotraumatol.* 2020;11(1): 1729633. 10.1080/20008198.2020.1729633 32284821 PMC7144187

[ref-45] Liberzon I Krstov M Young EA : Stress-restress: effects on ACTH and fast feedback. *Psychoneuroendocrinology.* 1997;22(6):443–453. 10.1016/s0306-4530(97)00044-9 9364622

[ref-93] Liu R Menhas R Saqib ZA : Does physical activity influence health behavior, mental health, and psychological resilience under the moderating role of quality of life? *Front Psychol.* 2024;15: 1349880. 10.3389/fpsyg.2024.1349880 38529092 PMC10961448

[ref-78] Lokshina Y Sheynin J Vogt GS : Fear extinction learning in Posttraumatic Stress Disorder. In: M. R. Milad & S. D. Norrholm (Eds.), *Fear Extinction: From Basic Neuroscience to Clinical Implications.*Springer International Publishing. *Curr Top Behav Neurosci.* 2023;257–270. 10.1007/7854_2023_436 37535308

[ref-46] Marsicano G Wotjak CT Azad SC : The endogenous cannabinoid system controls extinction of aversive memories. *Nature.* 2002;418(6897):530–534. 10.1038/nature00839 12152079

[ref-47] McAuley E Blissmer B : Self-efficacy determinants and consequences of physical activity. *Exerc Sport Sci Rev.* 2000;28(2):85–88. 10902091

[ref-48] Moya NA Tanner MK Smith AM : Acute exercise enhances fear extinction through a mechanism involving central mTOR signaling. *Neurobiol Learn Mem.* 2020;176: 107328. 10.1016/j.nlm.2020.107328 33075479 PMC7718627

[ref-49] Najavits LM : The problem of dropout from "gold standard" PTSD therapies. *F1000Prime Rep.* 2015;7: 43. 10.12703/P7-43 26097716 PMC4447050

[ref-80] Notarangelo FM Pocivavsek A Schwarcz R : Exercise your kynurenines to fight depression. *Trends Neurosci.* 2018;41(8):491–493. 10.1016/j.tins.2018.05.010 30053952

[ref-50] Notaras M van den Buuse M : Neurobiology of BDNF in fear memory, sensitivity to stress, and stress-related disorders. *Mol Psychiatry.* 2020;25(10):2251–2274. 10.1038/s41380-019-0639-2 31900428

[ref-51] Oppizzi LM Umberger R : The effect of physical activity on PTSD. *Issues Ment Health Nurs.* 2018;39(2):179–187. 10.1080/01612840.2017.1391903 29319376

[ref-52] Page MJ McKenzie JE Bossuyt PM : The PRISMA 2020 statement: an updated guideline for reporting systematic reviews. *BMJ.* 2021;372: n71. 10.1136/bmj.n71 33782057 PMC8005924

[ref-53] Peters JL Sutton AJ Jones DR : Contour-enhanced meta-analysis funnel plots help distinguish publication bias from other causes of asymmetry. *J Clin Epidemiol.* 2008;61(10):991–996. 10.1016/j.jclinepi.2007.11.010 18538991

[ref-54] Pitts BL Whealin JM Harpaz-Rotem I : BDNF Val66Met polymorphism and posttraumatic stress symptoms in U.S. military veterans: protective effect of physical exercise. *Psychoneuroendocrinology.* 2019;100:198–202. 10.1016/j.psyneuen.2018.10.011 30388593

[ref-55] Powers MB Medina JL Burns S : Exercise augmentation of exposure therapy for ptsd: rationale and pilot efficacy data. *Cogn Behav Ther.* 2015;44(4):314–327. 10.1080/16506073.2015.1012740 25706090 PMC4464974

[ref-56] Price M Lancaster CL Gros DF : An examination of social support and PTSD treatment response during prolonged exposure. *Psychiatry.* 2018;81(3):258–270. 10.1080/00332747.2017.1402569 30020026 PMC6207452

[ref-57] Ramos-Sanchez CP Schuch FB Seedat S : The anxiolytic effects of exercise for people with anxiety and related disorders: An update of the available meta-analytic evidence. *Psychiatry Res.* 2021;302: 114046. 10.1016/j.psychres.2021.114046 34126464

[ref-58] Robert M : The Role of Exercise in Reducing PTSD and Negative Emotional States. 2018. 10.5772/intechopen.81012

[ref-59] Schünemann H Brożek J Guyatt G : The GRADE handbook. Cochrane Collaboration,2013.

[ref-60] Shamseer L Moher D Clarke M : Preferred Reporting Items for Systematic Review and Meta-Analysis Protocols (PRISMA-P) 2015: elaboration and explanation. *BMJ.* 2015;350: g7647. 10.1136/bmj.g7647 25555855

[ref-61] Sheehan KH Sheehan DV : Assessing treatment effects in clinical trials with the discan metric of the Sheehan Disability Scale. *Int Clin Psychopharmacol.* 2008;23(2):70–83. 10.1097/YIC.0b013e3282f2b4d6 18301121

[ref-79] Sheynin J Shind C Radell M : Greater avoidance behavior in individuals with Posttraumatic Stress Disorder symptoms. *Stress.* 2017;20(3):285–293. 10.1080/10253890.2017.1309523 28322068 PMC5490437

[ref-62] Sterne JA Hernán MA Reeves BC : ROBINS-I: a tool for assessing risk of bias in non-randomised studies of interventions. *BMJ.* 2016;355: i4919. 10.1136/bmj.i4919 27733354 PMC5062054

[ref-63] Stewart AM Ullmann JFP Norton WHJ : Molecular psychiatry of zebrafish. *Mol Psychiatry.* 2015;20(1):2–17. 10.1038/mp.2014.128 25349164 PMC4318706

[ref-64] Stubbs B Vancampfort D Rosenbaum S : An examination of the anxiolytic effects of exercise for people with anxiety and stress-related disorders: a meta-analysis. *Psychiatry Res.* 2017;249:102–108. 10.1016/j.psychres.2016.12.020 28088704

[ref-65] Szafranski DD Gros DF Menefee DS : Predictors of length of stay among OEF/OIF/OND veteran inpatient PTSD treatment noncompleters. *Psychiatry.* 2014;77(3):263–274. 10.1521/psyc.2014.77.3.263 25162134

[ref-94] Ustün B Kennedy C : What is "functional impairment"? Disentangling disability from clinical significance. *World Psychiatry.* 2009;8(2):82–5. 10.1002/j.2051-5545.2009.tb00219.x 19516924 PMC2691163

[ref-66] Vancampfort D Richards J Stubbs B : Physical activity in people with Posttraumatic Stress Disorder: a systematic review of correlates. *J Phys Act Health.* 2016;13(8):910–918. 10.1123/jpah.2015-0436 27144877

[ref-67] Vancampfort D Stubbs B Richards J : Physical fitness in people with posttraumatic stress disorder: a systematic review. *Disabil Rehabil.* 2017;39(24):2461–2467. 10.1080/09638288.2016.1226412 27628485

[ref-68] Vecchio LM Meng Y Xhima K : The neuroprotective effects of exercise: maintaining a healthy brain throughout aging. *Brain Plast.* 2018;4(1):17–52. 10.3233/BPL-180069 30564545 PMC6296262

[ref-69] Vesterinen HM Sena ES Egan KJ : Meta-analysis of data from animal studies: a practical guide. *J Neurosci Methods.* 2014;221:92–102. 10.1016/j.jneumeth.2013.09.010 24099992

[ref-70] Weathers FW Blake DD Schnurr PP : The Clinician-Administered PTSD Scale for DSM-5 (CAPS-5). 2013. Reference Source 10.1037/pas0000486 PMC5805662 28493729

[ref-71] Weiss DS Marmar CR : The impact of event scale—revised.In: *Assessing psychological trauma and PTSD.*The Guilford Press,1997;399–411. Reference Source

[ref-72] Whitworth JW Scioli ER Keane TM : Physical inactivity, cigarette smoking, and psychiatric comorbidity among veterans with Posttraumatic Stress Disorder. *Health Psychol.* 2022;41(3):169–177. 10.1037/hea0001174 35298209

[ref-73] Wilker S Pfeiffer A Elbert T : Endocannabinoid concentrations in hair are associated with PTSD symptom severity. *Psychoneuroendocrinology.* 2016;67:198–206. 10.1016/j.psyneuen.2016.02.010 26923850

[ref-74] Wilkins SS Melrose RJ Hall KS : PTSD Improvement associated with social connectedness in gerofit veterans exercise program. *J Am Geriatr Soc.* 2020;69(4):1045–1050. 10.1111/jgs.16973 33368144 PMC8209690

[ref-75] Wright S Furukawa TA Macleod MR : Mechanisms through which exercise reduces symptom severity and/or functional impairment in Posttraumatic Stress Disorder (PTSD): protocol for a living systematic review of human and non-human studies. 2023. 10.17605/OSF.IO/GXWJN PMC12102654 40416511

[ref-76] Yang L Wang J Wang D : Delayed behavioral and genomic responses to acute combined stress in zebrafish, potentially relevant to PTSD and other stress-related disorders: focus on neuroglia, neuroinflammation, apoptosis and epigenetic modulation. *Behav Brain Res.* 2020;389: 112644. 10.1016/j.bbr.2020.112644 32344037

[ref-77] Zalta AK Tirone V Orlowska D : Examining moderators of the relationship between social support and self-reported PTSD symptoms: a meta-analysis. *Psychol Bull.* 2021;147(1):33–54. 10.1037/bul0000316 33271023 PMC8101258

