# Supplementary material for: Mechanisms through which exercise reduces symptom severity and/or functional impairment in posttraumatic stress disorder (PTSD): Protocol for a living systematic review of human and non-human studies
Source: Wellcome Open Res. 2025 Apr 14;8:494. Originally published 2023 Oct 25. [Version 3] doi: 10.12688/wellcomeopenres.19903.3 (PMC12102654; doi:10.12688/wellcomeopenres.19903.3)
Supplement: Peer review history

# Reviewer report: Reviewer response for version 3

**DOI:** 10.21956/wellcomeopenres.26151.r122744

**Published:** 2025-05-23

**Authors:** Cecila Hillard

**Affiliations:** 1 Medical College of Wisconsin, Wisconsin, USA

This is a study protocol that will serve as a living systemic review of literature regarding mechanisms involved in exercise-induced changes in PTSD symptoms and severity.  It will include both human and animal studies and, as such, will be an excellent vehicle by which translationally consistent findings can be assessed.  I completely agree with the authors that the time for demonstrating that exercise has a positive effect is passed and it is now time to determine the mechanisms.

 I do not have any suggestions, I think that the background and justification are well done, and the inclusion and exclusion criteria are appropriate.

 This meta-analysis will be an excellent addition to the literature.

Is the study design appropriate for the research question?

Yes

Is the rationale for, and objectives of, the study clearly described?

Yes

Are sufficient details of the methods provided to allow replication by others?

Yes

Are the datasets clearly presented in a useable and accessible format?

Not applicable

Reviewer Expertise:

Research into endocannabinoid signaling in both PTSD and exercise.  I have participated in preclinical and human subjects studies

I confirm that I have read this submission and believe that I have an appropriate level of expertise to confirm that it is of an acceptable scientific standard.

# Reviewer report: Reviewer response for version 3

**DOI:** 10.21956/wellcomeopenres.26151.r122222

**Published:** 2025-04-25

**Authors:** Jony Sheynin

**Affiliations:** 1 Texas A&M University, Houston, Texas, USA

I appreciate all the work that the authors did during the revision; I have no further comments.

Is the study design appropriate for the research question?

Yes

Is the rationale for, and objectives of, the study clearly described?

Yes

Are sufficient details of the methods provided to allow replication by others?

Yes

Are the datasets clearly presented in a useable and accessible format?

Not applicable

Reviewer Expertise:

Research on psychopathologies and neurological conditions, with a focus on the following domains: (1) Studying neural patterns using fMRI; (2) Studying decision-making patterns using computer tasks; (3) Studying the cognitive mechanisms that underlie maladaptive processes using computational modeling.

I confirm that I have read this submission and believe that I have an appropriate level of expertise to confirm that it is of an acceptable scientific standard.

# Reviewer report: Reviewer response for version 2

**DOI:** 10.21956/wellcomeopenres.26012.r117139

**Published:** 2025-01-20

**Authors:** Jony Sheynin

**Affiliations:** 1 Texas A&M University, Houston, Texas, USA

- I suggest that the authors briefly mention the idea that exercise can help with building resiliency, which is a very relevant and important topic that could be reviewed separately.
- Re :“functional impairment”: What is mentioned on pg 7 (“e.g., disability, quality of life”) is not a sufficient definition. I suggest that the authors take the text they had in their response and insert it early in the manuscript.

Is the study design appropriate for the research question?

Yes

Is the rationale for, and objectives of, the study clearly described?

Yes

Are sufficient details of the methods provided to allow replication by others?

Yes

Are the datasets clearly presented in a useable and accessible format?

Not applicable

Reviewer Expertise:

Research on psychopathologies and neurological conditions, with a focus on the following domains: (1) Studying neural patterns using fMRI; (2) Studying decision-making patterns using computer tasks; (3) Studying the cognitive mechanisms that underlie maladaptive processes using computational modeling.

I confirm that I have read this submission and believe that I have an appropriate level of expertise to confirm that it is of an acceptable scientific standard, however I have significant reservations, as outlined above.

# Response

**Published:** 2025-01-31

**Authors:** Simonne Wright87

**Affiliations:** Department of Psychiatry, Stellenbosch University, Stellenbosch, Western Cape, South Africa

**I suggest that the authors briefly mention the idea that exercise can help with building resiliency, which is a very relevant and important topic that could be reviewed separately**.

***Author response *** To address the reviewer’s suggestion, we have revised the introduction to include the following: “ Furthermore, exercise has been shown to positively impact resilience and mental health across diverse populations (Lancaster & Callaghan, 2022; Liu et al., 2024). For example, a cross-sectional study conducted during the COVID-19 pandemic found that exercise, particularly outdoor group activities, enhanced resilience, with stronger effects observed in women (Lancaster & Callaghan, 2022). These findings highlight the potential of exercise as a valuable strategy for promoting resilience and mitigating the negative effects of traumatic events (Kochi et al., 2017).” (pg 7)  

**Re :“functional impairment”: What is mentioned on pg 7 (“e.g., disability, quality of life”) is not a sufficient definition. I suggest that the authors take the text they had in their response and insert it early in the manuscript**.

***Author response *** To address the reviewer’s request for a more precise definition of 'functional impairment' and to clearly distinguish it from symptom severity, we have added the following clarification in the introduction: “Considering the above, exercise can play an important role in reducing symptom severity and/or improving functional impairment (e.g., disability, quality of life) in patients with PTSD and other trauma-related mental disorders. Functional impairment captures the extent to which symptoms of PTSD interfere with daily activities. This outcome reflects the impact of PTSD on quality of life and day-to-day functioning and is distinct from symptom severity, as a person might experience high functional impairment even with moderate symptom severity, or vice (Ustün & Kennedy, 2020).” (pg 8)

# Response

**Published:** 2025-04-01

**Authors:** Simonne Wright87

**Affiliations:** Department of Psychiatry, Stellenbosch University, Stellenbosch, Western Cape, South Africa

**I suggest that the authors briefly mention the idea that exercise can help with building resiliency, which is a very relevant and important topic that could be reviewed separately**.

**Author response** To address the reviewer’s suggestion, we have revised the introduction to include the following: “Furthermore, exercise has been shown to positively impact resilience and mental health across diverse populations (Lancaster & Callaghan, 2022; Liu et al., 2024). For example, a cross-sectional study conducted during the COVID-19 pandemic found that exercise, particularly outdoor group activities, enhanced resilience, with stronger effects observed in women (Lancaster & Callaghan, 2022). These findings highlight the potential of exercise as a valuable strategy for promoting resilience and mitigating the negative effects of traumatic events (Kochi et al., 2017).” (pg 7)  

**Re :“functional impairment”: What is mentioned on pg 7 (“e.g., disability, quality of life”) is not a sufficient definition. I suggest that the authors take the text they had in their response and insert it early in the manuscript**.

***Author response*** To address the reviewer’s request for a more precise definition of 'functional impairment' and to clearly distinguish it from symptom severity, we have added the following clarification in the introduction: “Considering the above, exercise can play an important role in reducing symptom severity and/or improving functional impairment (e.g., disability, quality of life) in patients with PTSD and other trauma-related mental disorders. Functional impairment captures the extent to which symptoms of PTSD interfere with daily activities. This outcome reflects the impact of PTSD on quality of life and day-to-day functioning and is distinct from symptom severity, as a person might experience high functional impairment even with moderate symptom severity, or vice (Ustün & Kennedy, 2020).” (pg 8)

# Reviewer report: Reviewer response for version 1

**DOI:** 10.21956/wellcomeopenres.22038.r106656

**Published:** 2025-01-07

**Authors:** Courtney Bouchet

**Affiliations:** 1 Colorado State University, Fort Collins, Colorado, USA

In *Mechanisms through which exercise reduces symptom severity and/or functional impairment in posttraumatic stress disorder (PTSD): Protocol for a living systematic review of human and non-human studies*, the authors outline detailed methods for a living systematic review. This includes various methods for exclusion and inclusion of experiments examining PTSD or PTSD-like behaviors and phenotypes in human and non-human studies, respectively. The methods outline how the data will be extracted from individual publications and strategies to reduce bias. Ultimately, while this is an important avenue of research for the inclusion of exercise as a treatment option for PTSD, this publication is not contributing to the literature and does not warrant an independent publication. This would be a suitable supplement for the living review itself or could be modified to be substantially shorter and included within the review as the methods section.

Is the study design appropriate for the research question?

No

Is the rationale for, and objectives of, the study clearly described?

No

Are sufficient details of the methods provided to allow replication by others?

Yes

Are the datasets clearly presented in a useable and accessible format?

Not applicable

Reviewer Expertise:

Neurobiology of exercise and fear memory learning and memory

I confirm that I have read this submission and believe that I have an appropriate level of expertise to state that I do not consider it to be of an acceptable scientific standard, for reasons outlined above.

# Response

**Published:** 2025-01-24

**Authors:** Simonne Wright87

**Affiliations:** Department of Psychiatry, Stellenbosch University, Stellenbosch, Western Cape, South Africa

**In *Mechanisms through which exercise reduces symptom severity and/or functional impairment in posttraumatic stress disorder (PTSD): Protocol for a living systematic review of human and non-human studies*, the authors outline detailed methods for a living systematic review. This includes various methods for exclusion and inclusion of experiments examining PTSD or PTSD-like behaviors and phenotypes in human and non-human studies, respectively. The methods outline how the data will be extracted from individual publications and strategies to reduce bias. Ultimately, while this is an important avenue of research for the inclusion of exercise as a treatment option for PTSD, this publication is not contributing to the literature and does not warrant an independent publication. This would be a suitable supplement for the living review itself or could be modified to be substantially shorter and included within the review as the methods section.**

**Author response** Thank you for taking the time to review our protocol paper, *Mechanisms through which exercise reduces symptom severity and/or functional impairment in posttraumatic stress disorder (PTSD): Protocol for a living systematic review of human and non-human studies*. We respectfully disagree with the suggestion that the protocol paper does not warrant independent publication. Protocol papers are critical for maintaining transparency and accountability in systematic reviews. They provide a publicly accessible record of the planned methods and objectives, enabling readers to evaluate whether the living systematic review adheres to its original stated intent. This is particularly important for living reviews, where iterative updates are expected as new evidence emerges. Since the decision on whether protocols are suitable for publication in a journal depends on the journal's policies, it is important to highlight the policy of Wellcome Open Research regarding systematic review protocols: “The peer review focuses on whether the paper has full academic merit, not on interest or novelty, and we welcome protocols for any study design in these Wellcome-funded areas… Protocols for systematic reviews should be registered and must follow the PRISMA-P guidelines.” ( https://wellcomeopenresearch.org/for-authors/article-guidelines/preparing-a-study-protocol-article) Publishing a protocol separately ensures that the review process is grounded in pre-specified plans, reducing the potential for selective reporting or unintentional deviations. It provides a detailed roadmap of the methods, including inclusion and exclusion criteria, data extraction strategies, and bias mitigation measures, thereby enhancing the rigor and credibility of the review. The protocol paper also facilitates replication by making the methodological framework accessible to other researchers providing a replicable model for future systematic reviews in PTSD and related fields. Furthermore, publishing the protocol allows for early engagement with peers, enabling constructive feedback that can improve the quality of the systematic review before its results are synthesized. This engagement aligns with the ethos of transparency and collaboration promoted by open-access platforms like Wellcome Open Research. Living systematic reviews are dynamic by nature, incorporating new evidence over time. A protocol serves as a stable reference point, ensuring that updates to the review remain aligned with the original methodological framework. Additionally, including an abridged version of the methods in the main review paper will not capture the depth of methodological detail or rationale that would be provided in a standalone protocol. Having a dedicated protocol allows readers to fully understand the rigor underlying the systematic review process. In light of these considerations, we believe that the independent publication of the protocol paper would significantly strengthen the transparency, reproducibility, and overall impact of our research. We hope this explanation underscores the importance of publishing the protocol in Wellcome Open Research.

# Reviewer report: Reviewer response for version 1

**DOI:** 10.21956/wellcomeopenres.22038.r106653

**Published:** 2024-11-06

**Authors:** Jony Sheynin

**Affiliations:** 1 Texas A&M University, Houston, Texas, USA

In this report, the authors outline a protocol for a living systematic review. The authors will review human and non-human literature on the mechanisms through which exercise reduces symptoms severity and functional impairment in PTSD. This is a very interesting topic with critical relevance to treatment of PTSD in particular, and mental conditions in general. Below I suggest edits that authors might want to consider to further improve this work:

- Currently, the focus of this work is on symptoms reduction. It would also be interesting and important to consider the role of exercise in resiliency; Can exercise make individuals more resilient and prevent/attenuate the development of PTSD symptoms after trauma exposure? In other words, from the perspective of PTSD symptomatology, is it beneficial to exercise only once symptoms are detected, or would it be beneficial to excise: 1) immediately at the aftermath of a traumatic event; or even 2) before any exposure to trauma occurrs? And if so, what are the possible underlying mechanisms?
- The concept of a “living review” is relatively novel. It would be helpful if authors explain this approach early in the manuscript.
- This protocol will focus on 2 main clinical outcomes: “symptom severity” and “functional impairment” (e.g., see title). While the first outcome is indeed very clear, I suggest to clarify the latter outcome. What is exactly meant by functional impairment in PTSD and how is it distinct from symptoms severity?
- The review will focus on individuals with PTSD. However, symptom severity and functional impairment can also be analyzed in subthreshold PTSD, i.e., in individuals who develop PTSD symptoms but are not diagnosed with PTSD, per se. This could also be included in this review and the title could be edited, accordingly.
- There is growing literature on the neural biomarkers involved in PTSD treatment, as detected by functional MRI (e.g., Rauch et al. 2018, PMID: 29081351). However, in this manuscript the research on neuroimaging/fMRI is not discussed. I suggest to elaborate on this topic, and state the key regions/networks that might be associated with the positive effects of exercise. 
- Introduction: When discussing regulation of fear extinction, it would be important to explain the role of fear extinction in PTSD (e.g., Lokshina et al. 2023, PMID: 37535308).
- Introduction: “Exercise has been reported to enhance pattern separation… which is important in differentiating safety from threat and restricting avoidance behavior”: Authors might want to briefly explain the greater avoidance behavior seen in PTSD (e.g., Sheynin et al. 2017, PMID: 28322068) and the overall relevance to impaired context processing (Liberzon & Abelson, 2016, PMID: 27710783).
- Pg. 5: “Research questions”: questions “1” and “2” in each section are very overlapping; Please revise and clarify. Related to this, on pg. 10 in “Data synthesis”, please make the described research questions align with their first presentation (e.g., are Research questions “1” focused on the role of mediators as stated on pg. 10? Please clarify).
- Figure 1: A visual demonstration of “effect modifiers” would be helpful.

**Minor:**

- Introduction: “people with PTSD are more likely than the general population to be physically inactive” — please include a citation.
- Subtitles “for non-human studies” and “for human studies”- consider removing “for” throughout the manuscript.
- Pg 5. For non-human studies: “…in animals with PTSD” — correct to “animal models of PTSD”.
- Methods: “Tri/Ab/MeSH” — please write full words, spell out and explain “MeSH”.
- Pg 8: Edit: “To gain a deeper understanding of research question II in humans, we will extract data on **impact of** exercise on…” 

Is the study design appropriate for the research question?

Yes

Is the rationale for, and objectives of, the study clearly described?

Yes

Are sufficient details of the methods provided to allow replication by others?

Yes

Are the datasets clearly presented in a useable and accessible format?

Not applicable

Reviewer Expertise:

Research on psychopathologies and neurological conditions, with a focus on the following domains: (1) Studying neural patterns using fMRI; (2) Studying decision-making patterns using computer tasks; (3) Studying the cognitive mechanisms that underlie maladaptive processes using computational modeling.

I confirm that I have read this submission and believe that I have an appropriate level of expertise to confirm that it is of an acceptable scientific standard, however I have significant reservations, as outlined above.

## References

[1] : Integrating biological treatment mechanisms into randomized clinical trials: Design of PROGrESS (PROlonGed ExpoSure and Sertraline Trial). *Contemp Clin Trials* .2018;64: 10.1016/j.cct.2017.10.013 128-138 10.1016/j.cct.2017.10.013 29081351

[2] : Fear Extinction Learning in Posttraumatic Stress Disorder. *Curr Top Behav Neurosci* .2023;64: 10.1007/7854_2023_436 257-270 10.1007/7854_2023_436 37535308

[3] : Greater avoidance behavior in individuals with posttraumatic stress disorder symptoms. *Stress* .2017;20(3) : 10.1080/10253890.2017.1309523 285-293 10.1080/10253890.2017.1309523 28322068 PMC5490437

[4] : Context Processing and the Neurobiology of Post-Traumatic Stress Disorder. *Neuron* .2016;92(1) : 10.1016/j.neuron.2016.09.039 14-30 10.1016/j.neuron.2016.09.039 27710783 PMC5113735

# Response

**Published:** 2025-01-06

**Authors:** Simonne Wright87

**Affiliations:** Department of Psychiatry, Stellenbosch University, Stellenbosch, Western Cape, South Africa

**In this report, the authors outline a protocol for a living systematic review. The authors will review human and non-human literature on the mechanisms through which exercise reduces symptoms severity and functional impairment in PTSD. This is a very interesting topic with critical relevance to treatment of PTSD in particular, and mental conditions in general. Below I suggest edits that authors might want to consider to further improve this work:**

*Authors’ response* Thank you for your thoughtful suggestion. We agree that understanding the role of exercise in building resilience and potentially preventing PTSD is an important avenue for research. However, we have chosen to focus specifically on populations with a clinical diagnosis of PTSD or those who have above-threshold symptoms of PTSD. This decision aligns with our goal to maintain a focused and clinically relevant scope that addresses the unique therapeutic needs and mechanisms pertinent to individuals already experiencing PTSD. The mechanisms underlying the effects of exercise any mirror mechanisms underlying PTSD, such as those involved in fear extinction, neuroplasticity, and stress response regulation. These mechanisms are often trauma-specific, with distinct neurochemical and physiological responses that may not be present in healthy individuals or those without a PTSD diagnosis. By examining these mechanisms within a PTSD cohort, we aim to better elucidate how exercise interventions can be optimized for therapeutic impact in this population.  

**The concept of a “living review” is relatively novel. It would be helpful if authors explain this approach early in the manuscript.**  

*Authors’ response* In the start of the Methods section, we have added: “ A LSR is a systematic review that is continually updated as new research emerges, ensuring the evidence base remains up-to-date. This method is particularly valuable in rapidly advancing fields, where integrating new findings in real-time can significantly improve clinical practice and policy-making (Elliott et al., 2014)” (pg 8) Additionally, under *Updating the systematic review and stopping the living mode of the review*, we state: “This study will be set up as an LSR, a high-quality online summary of the scientific literature updated as new research that meets study inclusion criteria becomes available (Elliott et al., 2014). Once we have completed the first version of the systematic review, the methods applied will be reconsidered to judge their suitability and efficiency in practice. If judged to be appropriate, the methodological approaches of data extraction and synthesis will be amended accordingly, and any changes will be documented. The literature search will be updated every three months, and any new studies meeting the inclusion criteria will be incorporated into the latest version of the evidence. If sufficient new data exists, a triangulation meeting will be held to incorporate the new evidence. Furthermore, it will be decided whether the review updates will continue, and whether to widen the scope of the review (this may include contacting authors of major studies to provide missing data). When there is insufficient new data to warrant triangulation, the review will be updated again after three months. We will also consider whether to incorporate hypothesis testing of identified probable mediators as a specific aim going forward. The living systematic review will use a versioning system based on the one used by F1000. We will clearly report all updates, and any deviations from the methods outlined in this protocol will be documented and justified.”  

**This protocol will focus on 2 main clinical outcomes: “symptom severity” and “functional impairment” (e.g., see title). While the first outcome is indeed very clear, I suggest to clarify the latter outcome. What is exactly meant by functional impairment in PTSD and how is it distinct from symptoms severity?**

*Authors’ response* Functional impairment captures the extent to which these symptoms interfere with daily activities. This outcome reflects the impact of PTSD on quality of life and day-to-day functioning and is distinct from symptom severity, as a person might experience high functional impairment even with moderate symptom severity, or vice versa. In our review, functional impairment will be assessed through validated scales like the Sheehan Disability Scale (SDS) and other self-reported or clinician-assessed measures that evaluate disability and quality of life. By including this outcome, we aim to provide a more comprehensive view of how exercise interventions may improve not only the symptoms of PTSD but also the overall functional capacity and life satisfaction of individuals affected by the disorder. In the methods section, under *Outcomes and prioritization*, we define functional impairment: “…functional impairment (e.g., disability, quality of life)…” (pg 7) **T**

**he review will focus on individuals with PTSD. However, symptom severity and functional impairment can also be analyzed in subthreshold PTSD, i.e., in individuals who develop PTSD symptoms but are not diagnosed with PTSD, per se. This could also be included in this review and the title could be edited, accordingly.**

*Authors’ response* We are including participants with above-threshold PTSD symptoms, in addition to those with a formal diagnosis of PTSD. Individuals with above-threshold PTSD symptoms on standardized self-report measure experience a clinically meaningful level of distress and impairment, even if they do not have a formal PTSD diagnosis. These participants often require similar treatment interventions and face comparable challenges in daily life as those with a formal diagnosis. By including them, the meta-analysis can provide insights that are applicable to a broader spectrum of individuals who would potentially seek or benefit from PTSD treatments in real-world clinical settings. Studies of PTSD treatments often use standardized inclusion criteria that require above-threshold symptoms or a formal PTSD diagnosis. Including below-threshold cases would likely introduce inconsistencies, as these cases might not meet the standard inclusion criteria in most trials. This approach keeps the meta-analysis consistent with the typical criteria used in PTSD research, enhancing the comparability and reliability of the findings.  

**There is growing literature on the neural biomarkers involved in PTSD treatment, as detected by functional MRI (e.g., Rauch et al. 2018, PMID: 29081351). However, in this manuscript the research on neuroimaging/fMRI is not discussed. I suggest to elaborate on this topic, and state the key regions/networks that might be associated with the positive effects of exercise.**

*Authors’ response* Under *Potential mediators* we stated: “… neuroimaging markers (e.g., MRI brain morphometry and function)…” (pg 8)  

**Introduction: When discussing regulation of fear extinction, it would be important to explain the role of fear extinction in PTSD (e.g., Lokshina et al. 2023, PMID: 37535308).** *Authors’ response* Added to the introduction: “ Fear extinction, essential for reducing fear responses to trauma-related cues, is often impaired in individuals with PTSD, resulting in persistent fear and anxiety (Lokshina et al., 2023) . These impairments, linked to dysfunctional neural circuits, especially in the amygdala and prefrontal cortex, hinder the retention of extinction memories, sustaining symptoms such as hypervigilance and intrusive thoughts. By helping individuals distinguish between trauma memories and safe present contexts, fear extinction can reduce anxiety and avoidance behaviors in everyday life.” (pg 6)  

**Introduction: “Exercise has been reported to enhance pattern separation… which is important in differentiating safety from threat and restricting avoidance behavior”: Authors might want to briefly explain the greater avoidance behavior seen in PTSD (e.g., Sheynin et al. 2017, PMID: 28322068) and the overall relevance to impaired context processing (Liberzon & Abelson, 2016, PMID: 27710783).**

*Authors’ response* Thank  you, we have updated to reflect your suggestion: “Further, exercise has been reported to enhance pattern separation and episodic memory (Déry et al., 2013), which is important in differentiating safety from threat and restricting avoidance behaviour. Avoidance behavior, a hallmark symptom of PTSD, reflects an impaired ability to process contextual cues, leading to the generalization of fear responses to non-threatening situations (Sheynin et al., 2017). It has been postulated that this effect is modulated by exercise-induced increases in hippocampal volume (Erickson et al., 2011).” (pg 7)  

**Pg. 5: “Research questions”: questions “1” and “2” in each section are very overlapping; Please revise and clarify. Related to this, on pg. 10 in “Data synthesis”, please make the described research questions align with their first presentation (e.g., are Research questions “1” focused on the role of mediators as stated on pg. 10? Please clarify).**

*Authors’ response* Research Question I is explicitly focused on understanding the mechanisms—specifically, the mediators that could explain how exercise may influence symptom severity and functional impairment in PTSD. Research Question II, on the other hand, aims to evaluate the direct effects of exercise itself on these outcomes. It is concerned with quantifying and understanding overall effects, rather than unpacking the pathways or mediators involved. This is stated in our research questions: “I. How does exercise reduce symptom severity and/or functional impairment in PTSD? II. What are the effects of exercise on symptom severity and functional impairment in individuals with PTSD, and which factors can moderate it? " (pg 5) Our data analysis section is in line with the research questions: " **Data synthesis** Research questions I: Exploring the role of mediators  Comparison of within-study direct and indirect effects A _ind_ and A _direct_ obtained from mediation analysis gives insight into the importance of the examined mediator. We will collect all study-specific estimates of the effects of type B, C, A _ind_ and A _direct_ and will group them by mediator. As, B and C might not have been calculated from proper mediation analyses, A _ind_ and A _direct_ would be only crude estimates of the true direct and indirect effects of exercise on the outcome. If enough estimates of sufficient quality are extracted for the same mediator, these can be synthesized across studies using random-effects meta-analyses (Cheung, 2022). Research question II: The impact of exercise on PTSD symptoms and function impairment  We expect several estimates of type A total to be available for potential synthesis from human studies. Heterogeneity will be assessed by visually inspecting forest plots considering the direction and magnitude of effects and the degree of overlap between Cis. If the heterogeneity is not judged to be substantial, we will conduct random-effects meta-analysis using the restricted maximum likelihood estimator of the between-study variance (τ 2). We will the Hartung-Knapp correction for the confidence intervals of the summary estimate when at least five studies are available. Heterogeneity will be quantified using the τ 2 and presented with the 95% prediction intervals (95% PI) of the treatment effects. When a meta-analysis is not feasible, we will synthesize the data without an accompanying meta-analysis (SwiM) ( Campbell et al., 2020). Furthermore, for non-human studies, we will conduct multilevel multivariable meta-regression with robust variance estimation (RVE)." (pg 17) We changed moderators to mediators under summary of evidence on page 19: “For research questions I, a SoE on Moderators mediators will provide: A list of variables that have been examined as mediators in the included studies…” (pg 18)

**Figure 1: A visual demonstration of “effect modifiers” would be helpful.**

*Authors’ response* Thank you for your thoughtful suggestion. After careful consideration, we would like to clarify that the primary focus of our review is on examining mediators of the effect of exercise on PTSD-related outcomes. In this context, effect modifiers found of the effect of exercise on PTSD-related outcomes will primarily be used to explore potential heterogeneity in the observed relationships using subgroup analysis and meta-regression.  

**Minor:** **Introduction: “people with PTSD are more likely than the general population to be physically inactive” — please include a citation.**

*Authors’ response* Thank you , we have added a relevant citation: “people with PTSD are more likely than the general population to be physically inactive (Vancampfort et al., 2016) ” (pg 6)  

**Subtitles “for non-human studies” and “for human studies”- consider removing “for” throughout the manuscript.**  

*Authors’ response* We have removed ‘for’ in the subheadings.  

**Pg 5. For non-human studies: “…in animals with PTSD” — correct to “animal models of PTSD”.**

*Authors’ response* Thank you, we have updated as suggested: “I. How does exercise manipulation change stress-related behaviours indicative of animal models of PTSD? PTSD in animals with PTSD?” (pg 8)  

**Methods: “Tri/Ab/MeSH” — please write full words, spell out and explain “MeSH”.** *Authors’ response* We have update accordingly: “In this first iteration, we will use a conventional search strategy ( Title [Ti], Abstract [Ab], Keywords and Medical Subject Headings [MeSH] Ti/Ab/Keyword/MeSH) … MeSH is a comprehensive controlled vocabulary used by the National Library of Medicine to index articles for PubMed, enabling precise and consistent retrieval of information on specific medical topics.” (pg 12)  

Pg 8: Edit: “To gain a deeper understanding of research question II in humans, we will extract data on **impact of** exercise on…”

*Authors’ response* Thank you for noting this oversight, we have updated to: “To gain a deeper understanding of research question II in human studies, we will extract data on impact of exercise on A Total for PTSD symptom severity and functional impairment…” (pg 14)
